# In memoriam: David G. Robinson

**DOI:** 10.1007/s00709-025-02059-9

**Published:** 2025-04-07

**Authors:** David Scheuring, Stefan Hillmer, Karin Schumacher

**Affiliations:** 1 Plant Pathology, University of Kaiserslautern-Landau, Kaiserslautern, Germany; 2 Cell Biology, Centre for Organismal Studies (COS), Heidelberg University, Heidelberg, Germany

**Keywords:** Biologist, North Shields, ENPER meetings

## Abstract

We are deeply saddened to report that David Gordon Robinson passed away on Tuesday, 5 November 2024. He has left behind his wife and three children. Without doubt, David was one of Europe’s leading plant cell biologists and electron microscopists, best known for his research on intracellular trafficking and cellular organization. He is leaving a legacy of groundbreaking research and influence in the field. In this obituary, we want to recapitulate the most important stages from the impressive career of a truly unique character.

## The early years

Born in the little British town North Shields in1947, David G. Robinson finished school in 1965 and started to study Biology at the University of Leeds. After university education, he obtained his PhD at the Astbury Department of Biophysics, also in Leeds, being only 25 years old. For his PhD thesis “The Synthesis and Orientation of Cellulose Microfibrils in Living Systems,” David employed the so-called freeze-etching technique in combination with electron microscopy on different algal cells. By this, he could structurally characterize organelles including the Golgi apparatus and chloroplasts as well as the cell wall (Robinson and Preston 1971a, b; Robinson 1972). Together, this resulted in eight publications, several of them in prestigious journals such as *Journal of Experimental Botany* and *Journal of Cell Science*, which were all published between 1971 and 1972. This allowed David to find a position, initially as postdoc and later as lecturer, at the renowned Stanford University in the USA. Although he spent only 2 years at Stanford, he later described this time as a very happy and productive stretch of his life. In Stanford, he worked on the plant cell wall and fibril organization and orientation using electron microscopy, a technique he employed and loved until his retirement. In 1974, he accepted a group leader position at the University of Göttingen, setting the course for the rest of his research career but also for his private life in Germany.

## Getting himself a name and foundation of the ENPER meeting

David G. Robinson did his Habilitation in Botany at the Georg-August-University Göttingen in 1976 being 29 years old. While he still used algae as model organisms, he also added cell cultures and vascular plants to his portfolio and continued his prolific publication output (Robinson et al. 1976; Robinson and Cummins 1976). Already in 1977, he was appointed professor having to learn German within 1 year before giving lectures in his new language. Succeeding to learn German, an achievement he was proud of, did not stop him from publishing and between 1977 and 1987, he published more than 50 scientific articles, several of them nowadays cited more than hundred times (Ray et al. 1976; Joachim and Robinson 1984). Nevertheless, it was not easy for him to be accepted in the German plant science community since his temper, his rather special British humor, his seemingly aggressive style in discussions, and his rough competitive behavior were considered not to be professorial. Although some students were pushed back by this, others enjoyed his open-minded discussions and loved that he was not holding back critical comments and thoughts. He always regarded teaching, especially in electron microscopy, as being very important and he therefore spent long hours with students at the microscope making sure that they knew how to operate the microscope and interpret electron micrographs correctly. One of his early students, Stefan Hillmer, was so captivated by electron microscopy that he did not only became tenured in David’s group but eventually helped shaping the Electron Microcopy Core Facility in Heidelberg. Over the years, whenever Stefan Hillmer was not sharing his opinion, David would jokingly shout: “Stefan, you are fired,” turning it into a running gag between them.

During his time in Göttingen, David’s research focus shifted progressively more towards vesicle trafficking, sorting and transport of storage proteins and different transport routes within the cell. Here, he was focusing on endocytosis as entirely new concept in plant cells. The prevailing opinion back then was that the thick cell wall and the high turgor pressure in plant cells would not allow the uptake of larger molecules. Not only did he establish endocytosis as uptake mechanism in plants (Hillmer et al. 1990; Robinson and Hedrich 1991; Robinson et al. 1992) but together with Susanne Holstein spearheaded the molecular characterization of transport vesicles including clathrin-coated vesicles from the plasma membrane (Demmer et al. 1993; Holstein et al. 1994; Robinson et al. 1998; Movafeghi et al. 1999). This offered the chance for David to join forces with scientists from the medical school and the Max Plank Institutes in Göttingen who together founded the Collaborative Research Centre 523—*Intracellular Transport and Trafficking –*improving the funding situation of his lab remarkably*.* In 1996, he also created the *European Network for Plant Endomembrane Research* (ENPER) meeting which is since held every year in a different country and still is a major event for the whole plant endomembrane community. The meeting brings together many different researchers working on plant membranes from all over Europe and even around the world. The emphasis at the ENPER meetings has always been to give young researchers, PhD students and post-docs, the opportunity to present their work in front of a highly specialized audience (Aniento et al. 2021). This might have sometimes led to stressful situations for the students, but almost always progressed the presented project, and hence, plant science in general.

## The Heidelberg—Hongkong connection

At a relatively progressed age (he was 53), David moved to the Ruprecht-Karls University Heidelberg for a full professorship for *Zellenlehre* that was formerly headed by Prof. Eberhard Schnepf (Behnke 1996). David realized soon that the *Zellenlehre* was too small to be a vital unit on the competitive Heidelberg Campus and fused it with the Botanical Institute, being one of the founding directors of the Heidelberg Institute for Plant sciences (HIP). A few years later, the directors of HIP and Heidelberg Institute of Zoology then formed the Centre for Organismal Studies (COS) to further increase the visibility of organismal biology on the Heidelberg Campus. An active exchange between David’s group and the group of Prof. Liwen Jiang in Hongkong resulted over the years in an adjunct professorship at the Chinese University of Hongkong (CUHK) in 2005. During this fruitful collaboration, David and other lab members (David Scheuring, Peter Pimpl, Stefan Hillmer) spent several weeks each year at the CUHK for collaborative purposes and for teaching. This vivid exchange went on and allowed young Chinese researchers to come to Germany to learn different techniques and PhD students from Germany to visit Prof. Liwen Jiang s lab at the CUHK.

In terms of science, David had his most prolific and successful time in Heidelberg. He was one of the first plant cell biologists to combine structural microscopy with biochemical methods such as cell fractionation and molecular biology. This broad methodology made his lab unique in plant sciences. Using this combination, David contributed significantly to understanding how proteins are sorted and transported in plant cells. In his lab, COPI vesicles were detected at the Golgi apparatus for the first time in plants (Pimpl et al. 2000), and together with Giselbert Hinz, he could establish the dense vesicle pathway for transport towards the storage vacuole (Hohl et al. 1996; Hinz et al. 1999, 2007; Hillmer et al. 2001; Robinson et al. 2005). Also in his lab, Brefeldin A was established as *bona fide* inhibitor of secretion (Nebenführ et al. 2002; Ritzenthaler et al. 2002) which is used nowadays as standard drug to manipulate trafficking. In the later years of his time in Heidelberg, David was fascinated by the plant-specific nature and dynamics of Golgi apparatus and its relation to the endoplasmic reticulum (ER). He was one of the leading researchers to locally define ER exit sites (ERES) and ER import sites (ERIS) and their behavior in respect to vesicular transport to and from the Golgi (Yang et al. 2005; Bubeck et al. 2008; Lerich et al. 2012). For years, he was enthusiastically debating this mechanism with his “scientific sparring partner” Chris Hawes from the Oxford Brooks University, UK (Neumann 2019), never really coming to a consensus. However, since they highly appreciated each other, they, together with other leading plant cell biologists, published a review article, explaining their arguments in detail (Robinson et al. 2015). Other groundbreaking findings involve the identification of the multivesicular body (MVB) as prevacuolar compartment (Tse et al. 2004) and the dependence of plant endocytosis on clathrin coated vesicles (Dhonukshe et al. 2007). Together with his successor Karin Schumacher, David could furthermore establish the *trans*-Golgi network (TGN) as merging place of secretion and endocytosis (Viotti et al. 2010) and reveal that the trafficking from the TGN towards the vacuole does not require clathrin but involves maturation of the TGN into the MVB (Scheuring et al. 2011).

For almost all his publications, his beloved technique of electron microscopy was employed, but new techniques and methods like confocal laser-scanning microscopy and molecular work were also (sometimes hesitantly) integrated in his research portfolio. A famous statement from the early days of confocal microscopy was “If you have nothing to say, say it in colors,” which might not have won him many new friends. However, David’s somewhat provocative statements often hit weak spots and almost always led to fruitful discussions. He emphasized clarity and precision in scientific language and mentored many students and postdocs. In addition, despite his sometimes harsh comments, he truly cared about and supported his students and coworkers, providing advice, writing recommendation letters, and connecting them to potential new working places.

## The late years

After his retirement in 2012, David Robinson continued publishing for more than another decade. Initially, he finished all research projects still left from his active time and voiced his opinion about critical questions in endomembrane research (Robinson and Pimpl 2014; Robinson et al. 2016; Robinson 2016, 2018, 2020). Afterwards, he focused his attention on criticizing the field of “plant neurobiology,” which he always opposed. Together with various other colleagues, he voiced his opinion against anthropomorphizing plant behaviors until the very end (Taiz et al. 2019; Robinson et al. 2020, 2024). Although he did not travel a lot during the last years, he remained a cosmopolite, having strong connections to researchers all over the world. By many colleagues, he was described as a brilliant electron microscopist and stimulating and critical conversationalist. He will be missed greatly as a scientist and person.

## David G. Robinson’s research in numbers

David G. Robinson has published close to 300 research and review articles, book chapters, and comments. His publications are cited more than 18,500 times and his H-index at some points surpassed his age (> 77), an achievement he was proud of and which he did not (mockingly) forget to mention during private conversations.

## Condolences from his colleagues and friends

David was also for us in Prague an orientational personality of our field. His “disputatious personality” inevitably resulted in emotional relationships and he will be remembered with gratitude by Czech plant cell biologists. ENPER community, which we cherish so much, is his living legacy. Sincerely from Prague,

Viktor Zarsky, Czech Republic

I was shocked to hear the news about David. I first met him at a keystone meeting, probably in 1983, and dozens of times since then, also within many scientific collaborations. David liked to play the disputatious guy, especially with friends (importantly), and I always had the impression he liked to play that role more as an actor than anything else—he actually had many biographies of classic film actors at home—and with the good intention of stimulating lively, deep discussions. He was a generous, intelligent, and very stimulating colleague. And of course a fantastic electron microscopist. His setting up of the ENPER meetings, first in Gottingen, then in Heidelberg and eventually passing the organizer role to different colleagues all over Europe, has been a great enterprise that for many years set the European scientific community at the front line of global plant cell biology research. We will always be grateful for this.

Good bye David, good man.

Sandro Vitale, Italy

We have lost a dear colleague and friend. He has been an important force structuring our field in Europe and beyond for many years.

I first met him at a meeting he organized in Göttingen. Ever since he started to organize the ENPER meeting I met him at least once a year. I remember intense discussions he had on occasions with the late Chris Hawes and others. We also had collaborations in European projects including a common PhD student along with Sandro Vitale. David kept his interest in science after his retirement and published up to this year.

We kept contact mostly by e-mail since the covid years. I knew about his health problems, but he remained intellectually fit. I miss him.

Jean-Marc Neuhaus, Switzerland

These were great times; scientifically and personally; and David was at the heart of them.

I will remember him very fondly as a great scientist, as a passionate debater and as a friend.

Take care; all of you.

Jiri Friml, Austria

I was very sad to hear the news about David and to say goodbye to a great scientist and a very good friend. We had a long and fruitful scientific collaboration over the years, which included exchange of PhD students between the two labs. He came several times to Valencia, and I also visited him in Heidelberg, including a very nice weekend during his retirement. I will certainly miss David and his strong personality, many email exchanges and long phone calls, the last one after the last ENPER meeting, and specially his sense of humor. I will always remember how often we were laughing on the phone.

Goodbye, David

Fernando Aniento, Spain

It was very sad to receive the news of David’s passing. He was a brilliant electron microscopist and a devoted plant cell biologist right up until the last few months. Many of us often received David’s interpretations of both recent and older publications, which often sparked lively discussions and diverse opinions. While people did not always agree with him, his perspectives stimulated important conversations, and that was invaluable for our group. In my view, David’s vision and the creation of ENPER stand as his lasting contribution to all of us. What began as a small group, primarily of Europeans (I was an outlier in the early days), has grown into a vibrant, unofficial “society” of plant cell biologists worldwide. We are all deeply grateful to David for this, and we will forever remember him as the father of ENPER.

David will be greatly missed.

Natasha Raikhel, USA

I am still shocked and deeply saddened after hearing the news. I first met David in my first year at the University in Göttingen more than 30 years ago. His commitment to science and passionate teaching triggered my interest in cell biology. With his will to foster scientific exchange and collaborations, he founded our ENPER community, which has been a fruitful source for exchanging ideas and personal career development for many of us ever since. Our ENPER community has lost a strong advocate for fair competition and truthful science. I will never forget him as a teacher, mentor, and friend.

Peter Pimpl, China
